# NRF2 Is One of the Players Involved in Bone Marrow Mediated Drug Resistance in Multiple Myeloma

**DOI:** 10.3390/ijms19113503

**Published:** 2018-11-07

**Authors:** Chia-Hung Yen, Hui-Hua Hsiao

**Affiliations:** 1 Graduate Institute of Natural Products, College of Pharmacy, Kaohsiung Medical University, Kaohsiung 80708, Taiwan; chyen@kmu.edu.tw; 2 Center for Infectious Disease and Cancer Research, Kaohsiung Medical University, Kaohsiung 80708, Taiwan; 3 Department of Medical Research, Kaohsiung Medical University Hospital, Kaohsiung 80708, Taiwan; 4 Division of Hematology-Oncology, Department of Internal Medicine, Kaohsiung Medical University Hospital, Kaohsiung 80708, Taiwan; 5 School of Medicine, College of Medicine, Kaohsiung Medical University, Kaohsiung 80708, Taiwan

**Keywords:** NRF2, multiple myeloma, resistance, bone marrow microenvironment

## Abstract

Multiple myeloma with clonal plasma expansion in bone marrow is the second most common hematologic malignancy in the world. Though the improvement of outcomes from the achievement of novel agents in recent decades, the disease progresses and leads to death eventually due to the elusive nature of myeloma cells and resistance mechanisms to therapeutic agents. In addition to the molecular and genetic basis of resistance pathomechanisms, the bone marrow microenvironment also contributes to disease progression and confers drug resistance in myeloma cells. In this review, we focus on the current state of the literature in terms of critical bone marrow microenvironment components, including soluble factors, cell adhesion mechanisms, and other cellular components. Transcriptional factor nuclear factor erythroid-derived-2-like 2 (NRF2), a central regulator for anti-oxidative stresses and detoxification, is implicated in chemoresistance in several cancers. The functional roles of NRF2 in myeloid-derived suppressor cells and multiple myeloma cells, and the potential of targeting NRF2 for overcoming microenvironment-mediated drug resistance in multiple myeloma are also discussed.

## 1. Introduction

Multiple myeloma (MM), characterized with malignant clonal expansion of plasma cells in bone marrow with defective production of monoclonal gammopathy, is the second most common hematologic malignancy, which accounts for 13% of all hematologic malignancies and 1% of all cancers in the world [1,2]. It generally occurs in elderly individuals with a median age of 69 years old at diagnosis and a median overall survival of six to seven years, which hinders traditional chemotherapeutic agents due to comorbidities and poor performance status [3]. The disease is generally considered incurable; however, the overall survival rate has improved gradually in the past few decades from the previous median survival of around three years to about five years in recently diagnosed patients. A certain population of patients even have an expected survival of more than 10 years [4,5]. Survival results of the intensive treatment approach and novel agents, which achieve an effective and sustained response, have improved in recent years [2,6].

With the introduction of high dose chemotherapy with stem cell support for eligible patients and the development of numerous novel agents for MM patients, all these procedures have been considered as standard therapy and dramatically improved the outcomes of patients, even in elderly individuals [1,4,7,8]. Over the past 20 years, more than 10 agents in different classes have been approved and plenty of therapeutic protocols with different distinct mechanisms were developed [9].

Traditionally, chemotherapeutic agents, including alkylating agents (cyclophosphamide and melphalan) and anthracyclines (doxorubicin) are often prescribed, and are typically combined with glucocorticoids (prednisone, dexamethasone) for MM therapy. Current therapeutic strategies include a combination of proteasome inhibitors (PIs), such as bortezomib, carfilzomib, and ixazomib, and immunomodulatory drugs (IMiDs), such as thalidomide and lenalidomide, with glucocorticoid as a first line therapy [10,11]. Recently, novel agents in the immunotherapy field have also been developed with exciting achievements for myeloma patients. Monoclonal antibodies targeting Cluster of Differentiation (CD)38, CD139/signal lymphocytic activation molecule F7 (SLAMF7), and B cell maturation agents (BCMA) have been developed and are available for clinical use as a first line treatment and/or for the relapsed condition of myeloma patients [12,13,14,15]. Novel agents with epigenetics modifications, such as histone deacetylase inhibitors, checkpoint inhibitors, cell cycle inhibitors, and/or alterative approaching with immunotherapy, such as chimeric antigen receptor T (CAR-T) therapy, and novel antibodies are under development [16,17]. Most MM patients respond successfully to initial therapy, however, the disease eventually relapses and turns it into an incurable disease. The significant contributor to disease relapse and/or treatment failure is the emergence of drug resistance [18,19].

Basically, MM displays complicated karyotypes and a high level of genomic/chromosome instability associated with various gene mutations and chromosome translocation, which might contribute to drug resistance [20]. However, oncogenomics studies found only a few differences that distinguish malignant MM from monoclonal gammopathy of undetermined significance (MGUS), implying that genetic changes may not totally explain the mechanisms of disease progression and resistance [21,22]. Recent studies showed that modifications within bone marrow milieu may confer MM cell survival and resistance to therapeutic agents through the delivery of soluble factors, such as cytokines and chemokines [23], cell to cell interaction with blood cells, endothelial cells, stromal cells, osteoclasts, adipocytes [24,25], and neo-angiogenesis [26], and by metabolic changes, such as oxygen and nutrition withdrawal [27]. Moreover, new mechanisms have emerged to participate in the drug resistance of MM cells within the bone marrow microenvironment, such as dysregulation of non-coding RNA species, like miRNAs, epigenetics modifications [28,29], and other cellular components, such as exosomes or microvesicles [30].

These mechanisms, including chromosome abnormalities, gene mutations, modifications of the bone marrow microenvironment with soluble factors, and/or adhesion of components and cells, all work together to tune the signal pathways that suppress the immune response and promote MM proliferation, survival, and drug resistance. Thus, knowing these mechanisms also turns on a niche for a novel therapeutic strategy to overcome drug resistance for disease treatment [31,32,33,34]. In this review, we discuss the mechanisms of disease progression and drug resistance from bone marrow microenvironment milieus focusing on the components and cells that sustain MM plasma cells survival and proliferation, which contribute to MM resistance. Interestingly, increasing evidence suggested that oxidative stress or reactive oxygen species (ROS) content play vital roles in the remodeling of the bone marrow microenvironment and the primary function of myeloid cells [35,36]. Knowing the nuclear factor erythroid-derived-2-like 2 (NRF2) is the master regulator of ROS detoxification and associated drug resistance of cancer cells [37], this narrative review will also discuss the functional roles of NRF2 in bone marrow microenvironment components.

## 2. Dual Roles of Nuclear Factor Erythroid-Derived-2-Like 2 (NRF2) in Cancer Development and Therapy

In response to oxidative stress and xenobiotic exposure, the NRF2‒Kelch-like erythroid cell-derived protein with CNC homology (ECH)-associated protein 1 (Keap1) pathway induces the expression of cytoprotective genes and protects cells against oxidative stress and environmental toxicants [38]. NRF2 regulates the basal expression and manages the induction of genes encoding antioxidant and detoxifying enzymes, including heme oxygenase-1 (HO-1), NAD(P)H:quinone oxidoreductase-1 (NQO1), catalase, superoxide dismutase (SOD), glutamate cysteine ligase (GCLC), and glutathione *S*-transferase (GST) [39,40]. Under normal condition, NRF2 is bound to Keap1 in the cytoplasm as an inactive complex, which facilitates the ubiquitination of NRF2 [41]. Modification of Keap1 cysteine residues reduces the binding affinity with NRF2, which in turn results in a decreasing of NRF2 ubiquitylation and subsequent stabilization of NRF2. Accumulated NRF2 then translocates into the nucleus where it forms complexes with small musculoaponeurotic fibrosarcoma (Maf), binds to the antioxidant response elements (AREs), and enhances the transcription of downstream genes [39]. Due to its cytoprotective function, NRF2 has been traditionally considered as a tumor suppressor and a target for chemoprevention [42]. However, recent genetic analyses of human cancer revealed that NRF2 could have a tumor promoting ability and cause resistance to chemotherapy. Moreover, abnormally high expression of NRF2 is frequently seen in human tumor specimens and is correlated with poor prognosis in patients with lung cancer, colorectal carcinoma, and head and neck squamous cell cancer, as well as hepatocellular carcinoma [43,44,45,46]. In addition, evidence from studies in cancer cell lines and in mouse models supported the capability of NRF2 to enhance drug resistance against a diverse range of drugs, such as cisplatin, doxorubicin, 5-fluorouracil, paclitaxel, and etoposide [47,48,49,50].

Regarding the microenvironment, inflammation, a hallmark of cancer, has been shown to play critical roles in both the development and the progression of most cancers. Among the factors involved in inflammatory responses, the transcription factor-nuclear factor-κB (NF-κB) is one of the most important molecules that links chronic inflammation to cancer. Elevated IL-6 production and NF-κB activation contribute to cancer progression and chemo-resistance [51,52]. Interestingly, previous findings suggested a functional cross-talk between NRF2 and NF-κB signaling pathways. Depletion of NRF2 enhances NF-κB activity and subsequently increases the production of inflammatory cytokines, which suggests an anti-inflammatory role of NRF2 [53]. A recent review article described the tumor suppressive and tumor promoting effects of NRF2 in several hallmarks of cancer, underlining the potential of targeting this transcription factor for anticancer therapy [54].

## 3. Resistance from the Bone Marrow Microenvironment

Multiple myeloma is caused by neoplastic transformation of plasma cells from terminally differentiated B lymphocytes, which have the capability of immunoglobulin production [2,55]. Like normal plasma cells, malignant plasma cells mainly survive and proliferate in bone marrow milieu, which interact with surrounding bone marrow microenvironments physically and functionally for cell proliferation and survival [2]. The bone marrow microenvironment includes two compartments that will interact with MM cells, which promote cell proliferation and survival [56,57]. One is the non-cellular compartment formed by soluble factors, like cytokines, chemokines, and growth factors, and extracellular matrix proteins, like collagen, fibronectin, and laminin. The other compartment is a cellular part comprising of hematopoietic cells and non-hematopoietic cells, fibroblasts, osteoblasts, osteoclasts, endothelial cells, mesenchymal stem cells, myeloid-derived suppressor cells (MDSCs), and bone marrow stromal cells (BMSCs) [58,59].

The pathomechanisms of disease progression and resistance are complex and not fully understood [2,60]. However, the interaction between soluble factors, matrix proteins, and cellular compartments with MM cells confer survival, proliferation, and also drug resistance of therapeutic agents on MM cells [19,60,61]. The mechanisms of resistance in cancers due to bone marrow microenvironment effects are best explained majorly by two forms of environment factors, which are soluble factor-mediated drug resistance (SFM-DR) and cell-adhesion-mediated drug resistance (CAM-DR) [62,63] and in the MM situation. In addition to innate or de novo resistance from dys-regulated genetic/molecular pathways in MM cells and the bone marrow microenvironment, long-term exposure to therapeutic drugs also contributes to acquired resistance due to changes of drug metabolisms in MM cells or impaired DNA repair mechanisms [19]. These innate/de novo and acquired resistances interact with each other and make MM one of the most difficult malignancies in the world.

## 4. Soluble Factors of Drug Resistance

Various soluble factors have been noted to confer resistance of therapeutic agents to MM cells. Interleukin-6 (IL-6) could be one of the most critical factors for MM survival and proliferation, and also the best explanation of SFM-DR and has been implicated for resistance to various therapeutic agents, including bortezomib, glucosteroid, and other agents [58]. IL-6 can be secreted by BMSCs, MDSCs, and MM itself by an autocrine manner after the adhesion of MM cells with BMSC or fibronectin [64]. IL-6 binds to its receptor on the MM surface and activates the Janus kinase/Signal transducer and activator of transcription (JAK/STAT) signaling pathway, which have various effects on the apoptotic pathway, such as the Fas/Apo-1 ligand [65,66]. Primary MM samples with the capability of autocrine IL-6 production had greater resistance to dexamethasone than those without autocrine IL-6 production [67]. Additionally, blocking IL-6 receptors with the antibody, CNTO 328, increased the sensitivity of MM cells to bortezomib therapy [68]. Furthermore, with the trigger of IL-6 after adhesion of MM cell to stromal cells, the pro-survival signaling pathway, the NF-kB pathway, in stromal cells were activated, which supports MM cell survival and proliferation [30]. IL-6 also enhanced the secretion of vascular endothelial growth factor (VEGF), which promoted angiogenesis and played a part in the migration of MM cells [69,70]. While bortezomib inhibits the production of IL-6 and VEGF, secretion of IL-6 by stromal cells and/or MM cells leads to bortezomib resistance [71]. These observations highlight the crucial role of IL-6 in MM progression and survival and also their contribution to resistance.

IL-6 also participates in MM drug resistance by regulating transcription factors. JunB, a member of activator protein 1 family of transcription factor, is associated with MM progression with correlation of its expression and MM pathogenesis [72]. JunB expression in MM cells is mediated by soluble factors, particularly by IL-6. IL-6 activated JunB expression by either Mitogen-activated protein kinase/mitogen-activated protein kinase (MEK/MAPK) or NF-κB pathways with dose and time effects [72]. JunB supports MM cell survival, since JunB knockdown of MM cells by shRNA inhibited BMSC-induced plasma proliferation and increase of apoptosis when co-cultured with primary BMSC or stromal cell lines. Furthermore, with the use of monoclonal antibodies against IL-6 receptors, tocilizumab prevents JunB upregulation from BMSC effects. Therefore, modulation of JunB expression could promote MM cell proliferation and also contribute to drug resistance MM, which resulted from IL-6 expression in the bone marrow microenvironment [72].

Besides IL-6, there are many cytokines and factors that confer to SFM-DR, including insulin-like growth factor-1 (IGF-1) [73,74,75], hepatocyte growth factor (HGF) [76], stroma-derived factor-1 (SDF-1), VEGF, epithelial growth factor (EGF) [77], IL-8, and tumor necrosis factor-B (TNF-B) [78]. IGF-1 is produced by plasma cells and presented in the bone marrow microenvironment, where it promotes proliferation and drug resistance through activation of MAPK and phosphatidylinositol-4,5-bisphosphate 3-kinase (PI3K)/AKT signaling pathways [79]. It has been noted that the IGF-1/IGF-1R signaling pathway is constitutively activated in bortezomib-resistant MM cells lines, and that the serum concentration of IGF-1 is far higher in bortezomib-refractory patients than those who show a response to the drugs. Blocking IGF-1R or IGF-1 signaling could induce apoptosis in MM cells, which in turn has synergistic therapeutic effects in combination with bortezomib [73,74]. Furthermore, as a downstream target of IGF-1, AKT expression increases in response to proteasome inhibitors in pre-clinical MM studies and bortezomib resistance could be overcome by AKT inhibition in an early phase clinical trials [80].

HGF, another soluble factor, is also noted to be upregulated during MM progression and also enhances the expression of c-MET [81]. The signaling pathway is activated in MM cells and endothelial cells from relapse and refractory patients, which suggests its role of drug resistance. Using a c-MET inhibitor demonstrated inhibitory effects on endothelial cells from patients with refractory to bortezomib, and lenalidomide results in down regulation of angiogenic activity [81,82]. The c-MET inhibitor, tivantinib, has been used in a phase II trial, showing potential effects on relapse/refractory MM patients [83]. Enhanced by IL-6, VEGF secretion promoted angiogenesis of MM, which plays a role in migration of MM, which contributes to disease progression [69]. Downstream activation of epidermal growth factor receptor (EGFR) signaling and reduced expression of tight junction protein 1 (TJP1) are strongly correlated with bortezomib resistance [2,69,77].

## 5. Cell Adhesion Mediated Resistance

Interaction between MM cells and components in the bone marrow microenvironment by adhesion to each other provide protective effects from cytotoxic agents of MM therapy [84,85]. It is accepted that CAM-DR contributes drug resistance to melphalan, vincristine, doxorubicin, mitoxantrone, and bortezomib [86,87]. Aberrant expression of a variety of cell adhesion molecules on the surface of MM cells, which function as cell to cell and cell to extracellular matrix interaction through counter receptors could promote MM proliferation and survival and also confer resistance. These cellular interactions are mediated by the expression and activation of adhesion molecules. Integrin families, such as Very Late Antigen-4 (VLA-4), Syndecan-1 (CD138, SDC1), CD44, Vascular Cell Adhesion Molecule-1 (VCAM-1), Lymphocyte Function-Associated Antigen-1 (LFA-1), Mucin-1 antigen (MUC-1), and Intercellular Adhesion Molecule-1 (ICAM-1), have been shown to associate with CAM-DR [30,58].

VLA-4, a heterodimer of CD49d/α4 and CD29/β1 integrins on MM cells, facilitates the binding of myeloma cells to extracellular matrix components, such as fibronectin, by VCAM-1 [86,87,88,89]. Other integrin, including β7-integrin and VLA-5 (α5β1), also plays a role in CAM-DR of MM cells [90]. The ITGB7 gene, which encodes β7-integrin, is a transcriptional target of the MAF family, which is highly expressed in up to 50% of MM patients, particularly high in those carrying cytogenetic abnormalities of t(8;14), t(14;16), and t(14;20) due to IgH enhanced transactivation [91]. These adhesion components are believed to be associated with drug resistance, with the fact that primary MM cells with a higher expression of these adhesion molecules are more resistant to therapeutic agents. The resistance is believed to be selected during a treatment course, which contributes to acquired drug resistance by CAM-DR [92].

The acquired drug resistance is much more complex than de novo drug resistance and it takes time to acquire the resistance. A study comparing the gene profiles of isogenic cell lines with melphalan resistance by adhesion to fibronectin and by long-term exposure to melphalan revealed a change in the expression of 1479 genes in the acquired drug resistance group and only 69 genes in the de novo drug resistance group. It strongly indicates that CAM-DR is associated with post-transcriptional mechanisms while the acquired drug resistance is associated with specific transcriptomes’ change [93]. Thus, induction of β1-integrin, which causes VLA-4-mediated CAM-DR in MM, is characterized by G1 cell cycle arrest accompanied by an increase of expression in CDK inhibitors, p21^Cip1^/Waf1 and p27^Kip1^ [87], and a decrease in Bim (an apoptotic BCL-2 family) [94] and Cyclin A and Cyclin E activity [87]. These findings support the post-transcriptional mechanisms of CAM-DR, all of which is accomplished by the modulation of ubiquitin-dependent degradation mechanisms. Furthermore, adhesion of β1-integrin also induces resistance to apoptotic signals, such as Fas/Apo-1, in various hematologic malignancies, including MM, through post-transcription regulation mechanisms.

These integrin molecules interact with extracellular matrix components, such as fibronectin, which act as extrinsic factors to elicit intracellular responses that stimulate signaling pathways and cytoskeletal modification [85,95]. In addition, CAM-DR also elicits resistance through blocking a specific element of the caspase-mediated apoptotic pathway. As shown in the MM cell line, RPMI-8226, the study demonstrated direct inhibition of mitoxantrone-induced caspase-3 and caspase-7 cleavage [85]. Thus, through the binding of microenvironment components and integrin molecules, MM cells use many ways to develop resistance to cytotoxic therapeutic agents.

It is obvious that SFM-DR and CAM-DR act closely with each other and cannot be separated completely. The synergic effects of anti-apoptosis from the adhesion of fibronectin (β1 integrin of VLA-4) and IL-6 (gp130 signaling) was fully demonstrated in a study with activated STAT3 in MM cells [66]. Other studies have also demonstrated that treatment with HGF increased the adhesion effect to fibronectin mediated by VLA-4 integrin, which promotes drug resistance through PI3K and NF-κB signaling pathways [76]. These data demonstrated the close cross-talk between SFM-DR and CAM-DR in the complex bone marrow microenvironment to the resistance of MM.

## 6. Bone Marrow Stromal Cells and Resistance

In addition to soluble factors and adhesion mechanisms, many cellular factors within a bone marrow microenvironment also confer resistance to MM. Several types of cells, including BMSCs, fibroblasts, adipocytes, endothelial cells, and osteoblasts, are believed to build up the bone marrow stroma. Growth factors and cytokines are mainly produced by these cells both in normal and in pathological conditions [96]. Adhesion of MM cells to BMSCs can induce upregulation of Notch receptors on MM cells, which bind to specific ligands (Jagged) on BMSCs, which increases resistance due to anti-apoptosis mechanisms [94]. BMSCs also showed induced bortezomib-resistance NF-κB activity in MM cells, which was mediated by soluble factors, including IL-8 from BMSCs, which provides an explanation for bortezomib resistance in many MM patients [78].

Co-culture of BMSCs from MM patients with MM cell lines resulted in an increase of survivin that contributes to daunorubicin resistance in MM cells; and the resistance could be reversed by blocking survivin, which would decrease resistance to daunorubicin, melphalan, and dexamethasone agents, and inhibit the growth of MM cells [97,98]. As a receptor of hyaluronic acid (HA), CD44 confers resistance to lenalidomide in myeloma cells as the factor that CD44/HA adhered to BMSCs more strongly in lenalidomide-resistant MM cells, suggesting CD44 and BMSC-mediated CAM-DR [99]. Adhesion of BMSCs upregulated B7-H1 (PD-L1) molecules in MM cells and was associated with resistance to dexamethasone and melphalan in MM cells, with an increase of Bcl-2 and FasL levels [100]. PD-L1 has also been considered as an immune response evasion tool for malignant MM cells [101,102]. Adhesion of MM cells to BMSC and fibronectin upregulates heat shock protein-70 (HSP-70), which confers CAM-DR; blocking HSP-70 results in an increase of melphalan-induced apoptosis and reverses the CAM-DR [103].

In addition to BMSCs, there are many other cells in the bone marrow microenvironment that could interact with MM cells and contribute to drug resistance [104], including osteoclasts, MDSCs, macrophages, dendritic cells, mesenchymal stem cells, osteoblasts, adipocytes, and endothelial cells [19,57]. Osteoclasts come from the fusion of myeloid/monocytic precursor cells and response to osteoclastogenic factors, such as receptor activators of nuclear kappa B ligands (RANKL) and ILs [105]. Myeloma cells could promote osteoclast formation directly via endogenous expression of RANKL, tumor necrosis factor alpha (TNF-α), and macrophage inflammatory protein 1 alpha (MIP-1α) By endogenous expression of RANKL, tumor necrosis factor alpha (TNF-α), and macrophage inflammatory protein 1 alpha (MIP-1α), myeloma cells could promote osteoclast cell growth [106,107]. Cell to cell contact between MM cells and osteoclasts promote cell growth and survival and confer resistance to doxorubicin-induced apoptosis through secretion of IL-6 and osteopontin (OPN) from osteoclasts [108].

Macrophages have been implicated in the progression and metastasis of several cancers [109]. In a tumor microenvironment, it has the anti-tumor phenotype, which is characterized with the expression of TNF-α, IL-1, and IL-12. With the expression of IL-10, it also has the pro-tumor phenotype that contributes to the progression of disease by stimulating proliferation and angiogenesis, suppressing the immune system, and protecting against chemotherapy induced apoptosis [110]. In the MM environment, blood-derived monocytes are recruited to the marrow by myeloma derived c-x-c motif chemokine 12 (CXCL12) and become a pro-tumor macrophage phenotype [111]. The pro-tumor macrophages enhance myeloma cell proliferation and survival by secreting IL-6 and IL-10. It confers drug resistance to apoptosis induced by dexamethasone and melphalan by inhibiting the apoptotic caspase pathway and promoting angiogenesis [112,113]. Moreover, the interaction between macrophages and MM cells via P-selectin glycoprotein ligand-1 (PSGL1)/selectin and ICAM-1/CD18 activate extracellular signal–regulated kinase 1/2 (ErK1/2) and c-myc pathways and confer drug resistance in MM [114].

Adipocytes are responsible for fat storage in the body and make up a large component of bone marrow. In a healthy bone, the adipokines, leptin and adiponectin, have been shown to promote osteoblast differentiation, proliferation, and mineralization, but prevent osteoclastogenesis [115]. However, these adipokines also promote the growths of some solid tumors, such as breast cancer and prostate cancer [116,117]. The role of these adipokines in hematologic cancers is not well known, but co-culture of MM cell lines with adipocyte-containing media revealed soluble factors that could promote proliferation and migration of MM cells, while inhibiting apoptosis via leptin [118,119]. In addition, adipocytes also secrete high levels of IL-6 [120]. Furthermore, adiponectin has been associated with the reduction of myeloma cell survival by suppression of lipogenesis [121,122]. Recently, some studies showed adipocytes, such as chemerin, an adipokine from stromal cells and preadipocytes, were also found to associate with myeloma progression [123]. These data demonstrated the relationship between adipo-tissue in the marrow microenvironment with myeloma cells, and might be a therapeutic target for myeloma therapy in the future [124,125].

Dendritic cells are antigen-presenting cells that link innate and adaptive immunity by regulating T cell behavior. Compared to normal individuals, myeloma patients have more dysfunctional plasmoid dendritic cells with impaired T cell stimulatory abilities [126,127]. The immune tolerance of MM cells is partly mediated by IL-6 [128,129]. Dendritic cells may also influence myeloma growth directly, since the co-culture with dendritic cells and myeloma cells results in increasing clonogenicity. By inhibition of RANKL and A proliferation-inducing ligand (APRIL), a study showed a decrease of clonogenicity of dendritic cells [130]. Other myeloid cells involved in myeloma progression, such as megakaryocytes and eosinophils, are also involved in the survival of plasma cells by production of IL-6 and APRIL that promote the survival of myeloma cells [131].

## 7. Myeloid-Derived Suppressor Cells and Resistance

The presence of myeloid cells with immune-suppressive activity was first described by Duwe and Singhal in 1979 [132]. The term, MDSC, was formally introduced by Gabrilovich et al. to describe the heterogeneous population of immature myeloid cells, which expand during tumor progression in response to numerous tumor-derived soluble and exosome-bound factors [133]. MDSCs are generally defined as immature myeloid cells with the expression of CD11b and Gr1 surface markers in mice. Later studies revealed that two morphologically and phenotypically distinct subpopulations, M-MDSCs (similar to monocytes) and G-MDSCs (similar to granulocyte), can be further separated according to the expression profile of the Gr1 marker. The Gr1 marker is not a singular molecule, since antibodies that recognize GR1 actually bind to two epitopes, LY6G and LY6C, which are different molecules encoded by different genes. The use of LY6G and LY6C-specific antibodies allow the identification of two MDSC subsets: G-MDSCs (granulocytic MDSCs) have a CD11b^+^LY6G^+^LY6C^low^ phenotype, whereas M-MDSCs (monocytic MDSCs) are CD11b^+^LY6G^–^LY6C^hi^ [134,135,136]. In humans, classification of MDSCs is much more difficult than in mice. Since Gr-1 is absent in humans, the two subpopulations were identified by the following cell surface proteins: CD11b^+^CD33^+^CD14^+^CD15^−^HLA^−^DR^−/lo^ for M-MDSCs, and CD11b^+^CD33^+^CD14^−^CD15^+^HLA-DR^−/lo^CD66b^+^ for G-MDSCs. While the levels of these two populations are usually low in healthy individuals, they are frequently expanded in cancer patients [137,138]. In addition to these two subtypes, Solito et al. described a third subpopulation of putative MDSCs with the phenotype Lin^−^CD11b^+^CD33^+^CD14^−^HLA-DR^−^ and a promyelocytic morphology inversely correlated with the clinical response [139]. As the markers, such as CD11b, CD33, CD14, and CD15, commonly used for identifying human MDSCs are variably expressed on other myeloid cells, several other markers have also been used to describe human MDSCs [140,141]. However, none are sufficient and more investigation is still required for characterization and isolation of human MDSCs populations.

In multiple myeloma, the expansion of MDSCs have been reported by several studies [142,143,144,145]. Gorgun et al. reported that, in the peripheral blood and the bone marrow of active MM patients, the frequency of CD11b^+^CD33^+^CD14^−^CD15^+^HLA-DR^−/low^ MDSCs were significantly increased when compared with healthy donors. They also showed that MDSCs from MM patients processes the ability to support tumor cell growth and suppress T cell responses [143]. In addition, Favaloro et al. also reported a significant increase in G-MDSCs (CD11b^+^CD33^+^CD14^−^CD15^+^HLA-DR^−/low^) in the blood of patients with progressive MM. MDSCs from MM patients significantly induced the generation of regulatory T cells (Tregs) when compared with age-matched controls. However, the MDSCs isolated from both MM patients and age-matched controls were able to inhibit autologous T cell responses to a similar degree when assayed at the same concentration [145]. Together, these findings indicated multiple mechanisms might be working simultaneously, and contribute to the aggressiveness of MM.

The name of MDSC indicates their suppressive function in the tumor microenvironment, where MDSCs suppress the T cell-mediated anti-tumor immunity via inhibiting the function of effector T cells and antigen presenting cells [146,147]. Several different mechanisms have been reported to be utilized by MDSCs for suppressing the immune system [147]. In addition to induction of Tregs as mentioned above, MDSCs mediate T cell suppression through inducing the expression of arginase 1 (ARG1) [148,149], production of high levels of anti-inflammatory cytokines, such as TGF-β and IL-10 [150,151,152], and the generation of nitric oxide (NO) and ROS by enhancing inducible NOS (iNOS) and NADPH oxidase (NOX) [153,154].

The metabolism of l-arginine has been shown to play a critical role in the suppressive activity of MDSCs. MDSCs express high levels of both ARG1 and iNOS, which are enzymes that use l-arginine as a substrate. l-Arginine is converted to urea and l-ornithine by ARG1, while iNOS uses l-arginine to generate NO and l-citrulline. The enzymatic activities of these two enzymes result in the depletion of l-arginine. It is well documented that a shortage of l-arginine inhibits T cell function by blocking proliferation of T cells, down regulating T cell receptor (TCR) expression, and arresting the cell cycle in the G0 to G1 phase [155,156].

In addition to depleting l-arginine, iNOS suppresses the T cell response through production of NO. In the presence of ROS, NO can react with the superoxide ion (O_2_^−^) to form peroxynitrite (ONOO^−^), which is one of the most powerful oxidants that are generated in the organism. Peroxynitrites can react with cysteine, methionine, tryptophan, and tyrosine residues to induce nitration and nitrosylation of target proteins [157]. Associations between peroxynitrite production and tumor progression have been observed in many types of cancer, including multiple myeloma [158,159]. Bronte et al. reported that inhibition of ARG1 and iNOS activity pharmacologically reduced tyrosine nitration and restored tumor-infiltrating lymphocytes responsiveness to prostate cancer [160]. Moreover, Nagaraj et al. described a potential mechanism by which MDSC induced CD8^+^ T cell tolerance [154]. They demonstrated that MDSCs induced nitration of the TCR and CD8 molecules during direct contact with T cells by producing peroxynitrite. The nitration altered the specific peptide recognition of TCRs and caused the inability of CD8^+^ T cells to respond to antigen-specific stimulation. In addition to T cell receptors, nitration of C-C motif chemokine ligand 2 (CCL2) chemokine and STAT1 in splenocytes from tumor-bearing mice have been reported. Molon et al. showed that intratumoral peroxynitrite production caused nitration of CCL2, which hindered the tumor infiltration of T cells. Blockage of intratumoral peroxynitrite production enhanced T cell infiltration and tumor eradication [161]. Mundy-Bosse et al. found that MDSCs from tumor-bearing mice reduced interferon (IFN)-mediated phosphorylation of STAT1 on Tyr^701^ in splenocytes. An association between nitric oxide elevation and increased levels of nitration on STAT1 in splenocytes from tumor-bearing mice was observed. When compared with control tumor-bearing mice, splenocytes isolated from tumor-bearing iNOS knockout mice exhibited a significantly increased IFN response [162].

Previously studies suggested a central role of ROS played in the suppressive activity of MDSCs [158]. Elevated levels of ROS have been reported as a major characteristic of MDSCs isolated from both tumor-bearing mice and cancer patients [163,164,165]. The immune suppressive function of MDSCs isolated from tumor-bearing mice and patients with cancer can be abolished by inhibiting ROS production by MDSCs [163,164]. As iNOS contributes to the production of NO, the production of ROS by MDSCs was attributed to the expression of NADPH oxidase type 2 (NOX2) [166]. Activated NOX2 converts molecular oxygen to superoxide anions, which could be further converted to hydrogen peroxide, which then reacts with NO to produce peroxynitrite [167]. In activated phagocytes, ROS is mainly produced by NOX2, which is crucial for their antimicrobial function and for regulating inflammatory responses [168]. Previous studies have shown that, in MDSCs, NOX2 is required for ROS production, the ability to suppress T cell responses, and maintaining an immature status [169,170]. In addition, increased expression of NOX2 was observed in human MDSCs from normal donors co-cultured with tumor cell lines [171] and MDSC from tumor-bearing mice [170].

In addition to an immune suppressive function, MDSCs can support tumor growth and enhance drug resistance by secreting growth factors and cytokines [172]. Growing evidence suggests that a subpopulation of cancer cells called cancer stem cells (CSCs) or tumor initiating cells possess a tumor initiation ability and self-renewal capacity, and are responsible for recurrence and metastases [173]. Cui et al. reported that MDSCs isolated from ovarian cancer patients not only inhibited T cell activation, but also enhanced CSC gene expression, sphere formation, and cancer metastasis [174]. Among the growth factors and cytokines secreted by MDSCs, interleukin-6 (IL-6) is the central tumor growth factor in vitro and in vivo in MM [175], and has been verified to be a prognostic factor for MM [176]. Using a murine breast cancer cell model, Oh et al. showed that IL-6 production by MDSCs increased stemness, invasiveness, and distant metastasis of tumor cells [177]. Fan et al. showed that IL-6 signaling plays a critical role in maintaining the stemness like population and enhancing chemotherapeutic resistance of MM [178]. De Veirman et al. reported that inhibiting S100A9 did not affect MDSCs accumulation and activity; however, it reduced the secretion of IL-6 and IL-10 of MDSCs and suppressed MM growth [179]. Ramachandra et al. showed that MDSCs can protect MM cells from chemotherapy reagents, doxorubicin and melphalan; and, interestingly, this effect is mediated by soluble factors, such as IL-6 [180]. Together, it is possible that MDSCs promote tumor growth, and aggressiveness is mediated primarily though IL-6 [172]. Moreover, MDSCs could increase the survival of MM cells by activating the AMPK pathway and increasing the expression of anti-apoptotic factors, Mcl-1 and Bcl-2 [181]. Furthermore, Binsfel et al. demonstrated that MDSCs have even more diverging roles in MM. They observed an elevation of the level of angiogenesis-related factors in MM patient derived MDSCs and a pro-angiogenic effect of these cells by using chick chorioallantoic membrane assay [182].

## 8. Potential Roles of NRF2 in Cells in the Marrow Microenvironment

As mentioned previously, IL-6 could be one of the most critical factors for MM survival, proliferation, and drug resistance. Many cells in the bone marrow, including BMSCs, fibroblasts, MDSCs, macrophages, adipocytes, and dendritic cells, secrete IL-6. Elevated IL-6 levels in many kinds of cancer cells and in their tumor microenvironments were attributed to the activation of NF-κB signaling [51].

Interestingly, mutual inhibition of NRF2 and NF-κB were observed under several pathological conditions, including inflammation and cancers [183]. It has been demonstrated that NF-κB competes with NRF2 for the co-activator CREB-binding protein, (CBP)/p300, in the nuclei. In addition, NF-κB promotes the interaction of histone deacetylase 3 (HDAC3) with either CBP or Mafs, facilitating the recruitment of the co-repressor, HDAC3, to ARE, which in turn leads to deacetylation of histone and suppresses the expression of NRF2 target genes. Conversely, activated NRF2 suppresses NF-κB signaling [184,185]. In line with this notion, pharmacological activation of NRF2 had been shown to suppress IL-6 expression in several cell types. Pirfenidone and calycosin activate NRF2 signaling via different mechanisms and reduce IL-6 secretion in fibroblasts [186,187]. Red ginseng-derived saponin fraction decreases IL-6 production in macrophages and adipocytes in an NRF2 dependent manner [188]. In osteoblasts, glabridin reduces IL-6 secretion accompanied with elevated NRF2 expression [189]. In addition, macrophages and embryonic fibroblasts derived from NRF2-deficient mice showed greater activation of NF-κB and production of inflammatory cytokines, including IL-6 in experimental sepsis models [190].

**Figure 1:**
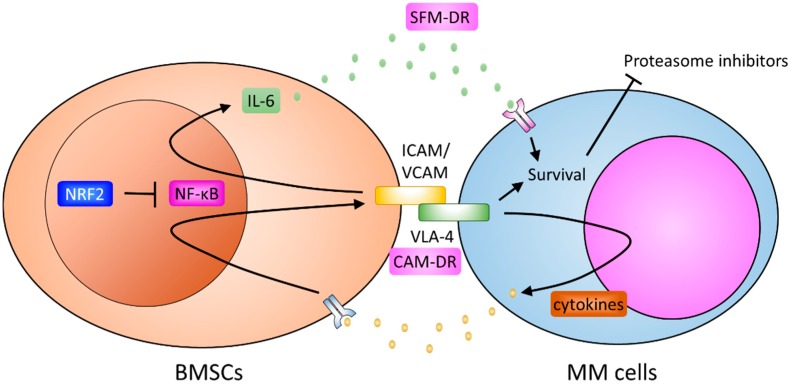
Roles nuclear factor erytheroid-derived-2-like 2 (NRF2) plays in bone marrow stromal cells (BMSCs). BMSCs could promote multiple myeloma (MM) progression through soluble factor-mediated drug resistance (SFM-DR) and cell-adhesion-mediated drug resistance (CAM-DR). MM cells could secrete cytokines/growth factors to induce the expression of adhesion molecules, such as intercellular Adhesion Molecule-1 (ICAM) and vascular Cell Adhesion Molecule-1 (VCAM), on BMSCs, and the secretion of cytokines, such as interleukin-6 (IL-6), from BMSCs. Interaction with BMSCs (CAM-DR) and/or stimulation by IL-6 (SFN-DR) could activate survival signals and result in the drug resistance of MM cells. Nuclear factor-κB (NF-κB) contributes to the expression of adhesion molecules and IL-6 in BMSCs. NRF2, through inhibiting the transcription activity of NF-κB, could repress both the SFM-DR and CAM-DR. Arrows indicate activation effects; T bars indicate suppressive effects.

It is of particular interest to note the adhesion molecule, VCAM-1, is also a target gene of NF-κB. Oncometabolite R-2-hydroxyglutarate induces the expression of VCAM-1 in bone marrow stromal cells via NF-κB signaling [191]. Suppression of NF-κB signaling in BMSCs reduces the expression of ICAM-1 and VCAM-1, and renders MM cells more sensitive to bortezomib treatment [192]. Likewise, activation of NRF2 could suppress the expression of these adhesion molecules in BMSCs by antagonizing the NF-κB pathway. Despite investigations of the effects of NRF2 on the expression of these adhesion molecules in BMSC being limited, activation of NRF2 has been associated with reduced expression of adhesion molecules in various pathological conditions. Curcumin, a well-known NRF2 activator, inhibits the expression of NF-κB and ICAM-1 in the middle cerebral artery occlusion rat model [193]. Dimethyl fumarate, or Tecfidera, elevates NRF2 and reduces pro-inflammatory cytokine production and VCAM-1 expression in the diabetic cardiomyopathy mouse model [194]. Isovitexin treatment effectively increases NRF2 expression while decreasing the expression of ICAM-1 and VCAM-1 in the lipopolysaccharide (LPS)-induced acute lung injury mouse model [195]. Together, these findings indicated that NRF2 could inhibit both the secretion of IL-6 and the expression of adhesion molecules in BMSCs; thus, reducing SFM-DR and CAM-DR (Figure 1).

Moreover, increased expression of iNOS, NOX2, and IL-6 in MDSCs is critical for them to exert tumor promoting functions. Previous studies indicated that NF-κB is a major regulator of LPS-induced iNOS expression in myeloid cells [196,197,198]. Moreover, Manea et al. showed that activation of NF-κB upregulated *p22^phox^* (a subunit of activated NOX2 protein) gene promoter activity [199]. In line with these observations, NF-kB has been shown to play a critical role in the accumulation and immune suppressive function of MDSCs [200,201,202].

In addition to NF-κB, activation of the JAK/STAT pathway plays a central role in regulating the inflammatory response. Activation of STAT3 was observed in MDSCs isolated from tumor-bearing mice. Conversely, inhibition of STAT3 reduced the expansion of MDSCs in tumor-bearing mice and reduced tumor progression [203,204]. A number of studies have shown that STAT3 participated in the regulation of iNOS, NOX2, and IL-6 expression in MDSCs [169,205,206]. Similar to NF-κB, STAT3 directly recruits transcriptional coactivators, CBP/p300, to promoters of STAT3 target genes, which in turn activate gene expression and/or alter chromatin structure [207,208].

**Figure 2:**
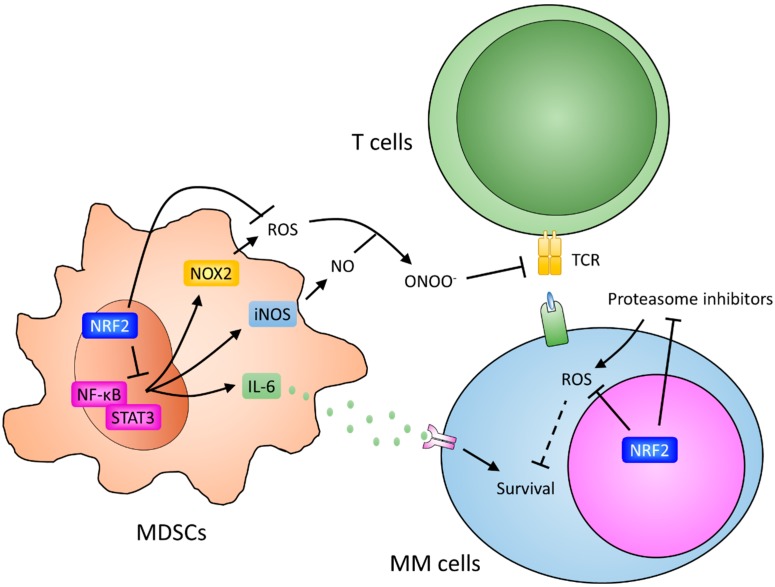
Roles NRF2 plays in MDSCs and MM cells. Myeloid-derived suppressor cells (MDSCs) could promote MM progression through immune suppressive activity and secreting cytokines, including IL-6. NF-κB and signal transducer and activator of transcription 3 (STAT3) contribute to the expression of iNOS and NOX2 in MDSCs. NO and ROS produced by iNOS and NOX2, respectively, will react with each other, then generate peroxynitrite (ONOO-). Peroxynitrite induced nitration of the T cell receptor (TCR) and CD8 molecules, which subsequently alter the specific peptide recognition and cause the inability of CD8+ T cells to respond to antigen-specific stimulation. On the other hand, IL-6 produced from MDSCs enhances proliferation and survival of MM cells directly. NRF2, through detoxification of ROS and inhibition of the transcription activity of NF-κB and STAT3, represses the immune suppressive function of MDSCs. However, NRF2 activation contributes proteasome inhibitors’ resistance in MM cells. Arrows indicate activation effects; T bars indicate suppressive effects.

These findings indicated that NRF2 could inhibit the immunosuppressive and tumor promoting functions of MDSCs through both inducing antioxidant gene expression and suppressing the expression of iNOS, NOX2, and IL-6 (Figure 2). In agreement with this notion, suppression of NRF2 has been shown to enhance the tumor promoting function of MDSCs. In mice studies, NRF2-deficiency creates a responsive microenvironment for pulmonary metastasis of the mouse Lewis lung carcinoma cells. As expected, high ROS levels were observed in the MDSCs isolated from tumor-bearing NRF2-deficient mice, which supports the notion that NRF2 inhibits the tumor promoting function of MDSCs by decreasing ROS production [209,210]. Interestingly, Kobayashi et al. recently reported that NRF2 could suppress the expression of IL-6 and IL-1β in an ROS-independent manner in myeloid cells [211], which supports our proposed model for the multiple functions of NRF2 in MDSCs (Figure 2).

Previously, bardoxolone methyl (also known as RTA-402, CDDO-methyl ester, and CDDO-Me), a potent synthetic triterpenoid compound, has been shown to be a potent NRF2 activator. A trial using RTA-402 in advanced pancreatic adenocarcinoma patients showed that RTA-402 did not alter the MDSC frequency in circulation. However, a significant increase in T cell responses to tetanus toxoid and phytohemagglutinin was observed in the RTA-402 treated group [212]. These studies led to the development of the second-generation triterpenoid drug, omaveloxolone (RTA-408). An ongoing phase 1b/2 clinical trial (NCT02259231) will evaluate the safety, efficacy, pharmacodynamics, and pharmacokinetics of RTA-408 in combination with Ipilimumab or Nivolumab in patients with unresectable or metastatic melanoma. Thus, it is worthwhile to elucidate the effect of RTA-402 or related compounds on the MDSC-mediated drug resistance of MM cells.

## 9. The Role of NRF2 in MM Cells

As mentioned above, NRF2 plays opposite roles in normal and cancer cells. While NRF2-deficiency enhances the tumor promoting function of MDSCs, recent studies demonstrated that NRF2 activation contributes to proteasome inhibitors resistance in MM (Figure 2). Yu Sun et al. reported that increased NRF2 activity, which activates pro-survival signaling pathways, was observed in primary MM and MM cell lines treated with bortezomib and carfilzomib. Ablation of NRF2 restored the sensitivity of MM cells to proteasome inhibitors [213]. Barrera et al. showed that bortezomib treatment induced the expression of HO-1, a target gene of NRF2, in time- and concentration-dependent manners. They also observed an elevated HO-1 level in lenalidomide-resistant MM cell lines [214]. Recently, Riz et al. [215] reported elevated protein expression of NRF2 and its activator, p62, in a carfilzomib-resistant MM cell. The increases of NRF2 and p62 protein resulted from EIF4E3 expression and activation of the PERK-eIF2α pathway, respectively. Genetic and pharmacologic inhibition of NRF2 or the PERK-eIF2α pathway restored the sensitivity to carfilzomib. Moreover, upregulation of EIF4E3 and NRF2 target genes were observed in chemoresistant and relapsed/refractory MM patients [215]. In addition, other studies also showed that the NRF2/POMP axis mediates bortezomib resistance in MM. Bortezomib-resistant myeloma cells overexpressed proteasome maturation protein (POMP). An association between overexpression of POMP and increased levels of NRF2 was also observed in bortezomib-resistant myeloma cells [216,217]. The NRF2 inhibitor, brusatol, has been shown to reduce the viability of MM cells [218]. However, the mechanism of action of brusatol is not through direct inhibition of NRF2 [219]. By high throughput screening of drug-like molecules, Liu et al. recently identified the CDK inhibitor, PHA-767491, as an NRF2 inhibitor, and suppressed MM cell growth [218].

New evidence has indicated that NRF2 not only is the master regulator of antioxidants and detoxification, but also plays roles in several cellular functions [220]. Thus, the Keap1-NRF2 pathway is an attractive therapeutic target for several diseases, including metabolic syndrome and cancers [221]. However, as we have discussed above, the functional roles of NRF2 could be opposite in BM environment cells and in MM cells. Thus, the effect of NRF2 activators on the growth and drug resistance of MM cells should be noted. Nonetheless, the fact that cancer cells can activate NRF2 through several mechanisms, including somatic mutations in *Keap1* or *NRF2* gene loci, hypermethylation at the promoter region of *Keap1*, transcriptional upregulation of the *NRF2* gene through oncogene-dependent signaling, interruption of Keap1-NRF2 interaction, and the modification of Keap1 protein by electrophilic oncometabolites, may provide possible resolution of this contradiction, in which cancer cells could lose their sensitivity to NRF2 activators [222,223]. In line with this notion, we found that cells, which expressed higher basal NRF2 protein levels, have a weaker response to NRF2 activators. Accordingly, NRF2 could be a potential target for treating bone marrow microenvironment-mediated MM drug resistance.

## 10. Interaction of Bone Marrow Microenvironment and MicroRNA

The recent development of the drug resistance of MM focused on the microRNAs for the pathomechanisms of bone marrow microenvironment induced drug resistance in recent studies [224]. miRNAs are short, single-strand, non-coding RNAs that target the 3′-UTR of mRNAs, preventing protein translation, and therefore regulating cell proliferation, metabolism, aging, and cell death [225]. With interaction of bone marrow stromal cells and certain kinds of miRNAs in MM cells, miRNAs can confer drug resistance depending on the positive or negative impacts of their targets [226,227]. miR-21 and miR-221/222 are known oncogenic miRNAs with high expression in several cancers and also in MM [228,229]. Overexpression of miR-21 suppresses its targets, such as phosphatase and tensin homolog (PTEN), PUMA, and RhoB, which promote MM viability and contribute to CAM-DR [228,230]. Interestingly, the expression of miR-21 after adhesion of MM and stromal cells are NF-κB dependent, as the NF-κB inhibitor, BAY 11-7082, prevents miR-21 upregulation in MM cells [231]. Elevated miR-221 was found in the plasma of MM patients [232]. Targeting miR-221 restores the sensitivity of MM cells to dexamethasone [233]. miR-27a was considered as a tumor suppressor in MM and leukemia as it targeted oncogenes, CDK5 and P-glycoprotein, which were highly expressed in tumor cells [234]. However, it was also associated with bortezomib resistance in MM [235]. miR-15a has been shown to be upregulated in MM cell lines when treated with bortezomib, and the upregulation of miR-15a by bortezomib treatment was suppressed when MM cells were co-cultured with bone marrow stromal cells, which implied the bortezomib drug resistance [71]. VEGF, as a target of miR-15a, is enhanced by miRNA-15a downregulation, and VEGF would stimulate IL-6 secretion by bone marrow stromal cells, providing a favorable environment for MM proliferation [236]. It is interesting to note that miR-27a can affect the expression level of the NRF2 protein [237]; and that miR-221/222 are potential miRNAs upregulated by NRF2 [238]. The expression of miR-202 is decreased in BMSCs from MM patients. Introduction of miR-202 mimics into BMSCs inhibits the NF-κB signaling pathway and reverses CAM-DR [192]. These findings provided insights into the mechanisms by which NRF2 participates in MM drug resistance.

## 11. Other Environmental Factors for the Resistance

The stress from oxygen and nutrition withdrawal and acidosis in bone marrow causes misfolded protein, chaperone dysregulation, enhanced ER stress, and activation of the unfolded protein response (UPR) [239]. The role of ER stress and UPR in MM has been demonstrated in several studies [239,240]. Three main sensors are known for stress, including kinase/endoribonuclease inositol requiring enzyme 1α (IRE1α), the kinase PKR-like endoplasmic reticulum knase (PERK), and the transcription factor activating transcription factor-6 (AFT-6). The PERK function mainly lowers protein translation and allows refolding activity of ER through inhibition of protein translations, except transcription factor 4 and NRF2 [241]. Hypoxia and the activation of UPR sustains malignant cell progression and resistance through the activation of intracellular and/or extracellular factors, including hypoxia-inducible factor-1α (HIF-1α), NRF-2, VEGF, IL-8, fibroblast growth factor-2 (FGF-2), and NF-κB, thus inducing metastasis, angiogenesis, and MM growth [59]. In addition, the role of infection/inflammatory environment of MM cells has been suggested by a wide range of functional Toll-like receptors (TLRs) in MM samples and cell lines [242]. Stimulating MM cells with TLR ligands induced secretion of IL-6, growth and proliferation of MM cells, upregulation of immune evasion markers, and differentially modulated expression of adhesion molecules and adhesion to fibronectin, which causes drug resistance [243]. In contrast, stimulation of some TLRs could also induce apoptosis in some MM cells and re-sensitize them to bortezomib in a fibronectin context. These also suggested the role of the environment of myeloma in myeloma drug resistance [242].

## 12. Concluding Remarks

Recent progress in the treatment of MM by novel agents makes a significant improvement in the outcome; however, in most of these cases, the disease progresses, relapses, and confers resistance to therapeutic agents [3]. Understanding of the mechanisms of disease progression and resistance in molecular/cytogenetic levels, soluble factors, cellular factors, and the bone marrow environment can be used to argue treatment strategies for MM survival and outcomes [244]. In addition to other mechanisms, the bone marrow microenvironment coping with soluble factors, adhesion factors, cellular factors, and environment stress plays an important role in disease progression and drug resistance. Through the interaction and cross-talk with these factors, these components orchestrate together and confer progression and resistance of MM by tuning the signaling pathways. Though the real mechanisms are complex and beyond our current understanding, identification of a variety of molecular and signaling pathways of drug resistance from components in the bone marrow environment has opened a new niche for novel targeted agents beyond conventional cytotoxic chemotherapy agents [245,246]. Finally, owing to the elusive and complex nature of MM, more research is urgently required for identifying novel agents targeting the bone marrow microenvironment components and/or dysregulated signaling pathways due to environment interaction.

## Abbreviations

MM	multiple myeloma
PIs	proteasome inhibitors
IMiDs	immunomodulatory drugs
CD	cluster of differentiation
SLAMF7	signal lymphocytic activation molecule F7
BCMA	B cell maturation agents
CAR-T	chimeric antigen receptor T
MGUS	monoclonal gammopathy of undetermined significance
ROS	reactive oxygen species
NRF2	nuclear factor erytheroid-derived-2-like 2
Maf	musculoaponeurotic fibrosarcoma
NF-κB	nuclear factor-κB
MDSCs	myeloid-derived suppressor cells
BMSCs	bone marrow stromal cells
SFM-DR	soluble factor-mediated drug resistance
CAM-DR	cell-adhesion mediated drug resistance
JAK/STAT	Janus kinase/Signal transducer and activator of transcription
IL-6	interleukin-6
VEGF	vascular endothelial growth factor
IGF-1	insulin-like growth factor-1
PI3K	phosphatidylinositol-4,5-bisphosphate 3-kinase
HGF	hepatocyte growth factor
SDF-1	stroma-derived factor-1
EGF	epithelial growth factor
EGFR	epidermal growth factor receptor
TJP1	tight junction protein 1
TNF-B	tumor necrosis factor-B
VLA-4	Very Late Antigen-4
SDC1	Syndecan-1
VCAM-1	vascular Cell Adhesion Molecule-1
LFA-1	lymphocyte Function-Associated Antigen-1
MUC-1	Mucin-1 antigen
ICAM-1	intercellular Adhesion Molecule-1
HA	hyaluronic acid
RANKL	receptor activator of nuclear kappa B ligand
HSP-70	heat shock protein-70
TNF-α	tumor necrosis factor alpha
MIP-1α	macrophage inflammatory protein 1 alpha
OPN	osteopontin
CXCL12	c-x-c motif chemokine 12
PSGL1	P-selectin glycoprotein ligand-1
Erk1/2	extracellular signal–regulated kinase 1/2
APRIL	proliferation-inducing ligand
Tregs	regulatory T cells
G-MDSCs	granulocytic MDSCs
M-MDSCs	monocytic MDSCs
ARG1	arginase 1
NO	nitric oxide
iNOS	inducible NOS
NOX	NADPH oxidase
TCR	T cell receptor
O_2_^−^	superoxide ion
ONOO^−^	peroxynitrite
CCL2	C-C motif chemokine ligand 2
IFN	interferon
CSCs	cancer stem cells
Keap1	Kelch-like ECH-associated protein 1
HO-1	heme oxygenase-1
NQO-1	NAD(P)H:quinone oxidoreductase-1
SOD	superoxide dismutase
GCLC	glutamate cysteine ligase
GST	glutathione S-transferase
AREs	antioxidant response elements
HDAC3	histone deacetylase 3
CBP	CREB-binding protein
POMP	proteasome maturation protein
PTEN	phosphatase and tensin homolog
ER	endoplasmic reticulum
IRE1α	kinase/endoribonuclease inositol requiring enzyme 1α
PERK	protein kinase RNA-like ER kinase
IRE1	inositol-requiring enzyme 1
ATF6	activating transcription factor 6
HIF-1α	hypoxia-inducible factor-1α
FGF-2	fibroblast growth factor-2
UPR	unfolded protein response
LPS	lipopolysaccharide
TLRs	Toll-like receptors
